# The Role of Periostin in Capsule Formation on Silicone Implants

**DOI:** 10.1155/2018/3167037

**Published:** 2018-04-26

**Authors:** Hahn-Sol Bae, Hye-Youn Son, Jung Pyo Lee, Hak Chang, Ji-Ung Park

**Affiliations:** ^1^Department of Plastic and Reconstructive Surgery, Seoul National University Boramae Medical Center, Seoul 07061, Republic of Korea; ^2^Department of Internal Medicine, Seoul National University Boramae Medical Center, Seoul 07061, Republic of Korea; ^3^Department of Plastic and Reconstructive Surgery, Seoul National University Hospital, Seoul 03080, Republic of Korea

## Abstract

Although silicone implants are widely used in breast and other reconstructive surgeries, the limited biocompatibility of these materials leads to severe complications, including capsular contracture. Here, we aimed to clarify the relationship between periostin and the process of capsule formation after in vivo implantation. Seven-week-old wild-type (WT) C57BL/6 mice and periostin-deficient mice were used. Round silicone implants were inserted into a subcutaneous pocket on the dorsum of the mice. After 8 weeks, the fibrous capsule around the implant was harvested and histologically examined to estimate capsular thickness and the number of inflammatory cells. Additionally, immunohistochemical analysis (periostin, *α*-SMA, and collagen type I) and western blotting (CTGF, TGF-*β*, VEGF, and MPO) were performed for a more detailed analysis of capsule formation. The capsules in periostin-knockout mice (PN-KO) were significantly thinner than those in WT mice. PN-KO mice showed significantly lower numbers of inflammatory cells than WT mice. Fibrous tissue formation markers (*α*-SMA, periostin, collagen type I, and CTGF) were significantly reduced in PN-KO mice. We also confirmed that inflammatory reaction and angiogenesis indicators (TGF-*β*, MPO, and VEGF) had lower expression in PN-KO mice. Inhibition of periostin could be important for suppressing capsule formation on silicone implants after in vivo implantation.

## 1. Introduction

The use of breast implants has gained attention due to the increased numbers of breast reconstructive and esthetic surgeries. Recently, almost 65% of women in the United States have chosen permanent implant-based reconstruction rather than autologous tissue for breast reconstruction after mastectomy [1]. In addition, esthetic breast augmentation constitutes approximately 20.8% of all plastic surgery procedures in the United States, and the number of cases continues to rise with increases in psychosocial as well as esthetic benefits [2].

However, complications may occur after silicone implant-based breast surgery, which include seroma, infection, bleeding, nipple sensory loss, scarring, and capsular contracture [3–6]. Among these, the most common and serious complication after implant insertion is capsular contracture. The incidence of capsular contracture ranges from 0.59% to 18.9% in women following various breast surgeries using silicone implants, including primary breast augmentation and postmastectomy breast reconstruction, or following revision surgery [7]. It has been reported that capsular contracture occurs over a time-scale ranging from several months to years after breast implantation [3, 8–10]. Despite these complications, the exact cause of capsular contracture has not been determined, although it has been considered to be a normal defense mechanism against a foreign body. When implants reside in the body for a prolonged period, fibrous connective tissue composed of collagen and fibroblasts accumulates around them as a natural response to the presence of a foreign body. In particular, an exaggerated foreign body reaction, which includes severe inflammatory and proliferative processes, results in excessive fibrotic tissue formation. Subsequently, a contractile force originating from collagenous capsulation causes capsular contracture around the implant [11, 12], eventually causing unbearable pain.

Periostin, also known as osteoblast-specific factor 2, is a secreted extracellular matrix (ECM) protein encoded by the 13q13.3 POSTN gene [13, 14]. This protein is a recently characterized matricellular protein that binds to components of the ECM, including type 1 collagen and fibronectin, and it has been shown to be involved in collagen fibrillogenesis [15]. Periostin protein transmits signals from the ECM to the cell by binding to cellular receptors such as integrins that affect cell adhesion, proliferation, migration, and tissue angiogenesis [14]. These roles of periostin are manifested in the processes of wound healing, fibrosis, and tissue regeneration. It has also been revealed that periostin promotes cancer cell invasion and metastasis through the integrin/phosphatidylinositol 3-kinase/AKT pathway, leading to tumor growth and metastasis [16–18]. Kyutoku et al. [19] previously reported that a periostin antibody inhibits the growth of breast and gastric cancers and fibrous tissue formation.

Although periostin has diverse functions, it is becoming increasingly clear that, in many cases, it is greatly involved in the progression of tissue fibrotic diseases such as scleroderma, which is a connective tissue disorder characterized by the excessive deposition of collagen and other ECM proteins that results in the fibrosis of skin and other visceral organs [20–24]. Mice with increased expression of periostin exhibit marked cutaneous sclerosis with increased numbers of myofibroblasts [25].

Capsular contracture is of particular interest in relation to the progression of tissue fibrosis.

In this study, we aimed to clarify the relationship between periostin and the process of capsule formation after in vivo silicone implantation. Although periostin is known to have a significant effect on tissue fibrosis, to the best of our knowledge, the present study is the first to reveal a relationship between periostin and capsular contracture.

## 2. Materials and Methods

### 2.1. Preparation of Animals

This study was approved by the Institutional Animal Care and Use Committee (IACUC) of the Seoul National University Boramae Hospital (IACUC number 2016-0056).

As experimental animals, we used 7-week-old male C57BL/6 and periostin-knockout (PN-KO) mice weighing 22 g (*n* = 6, each group). The PN-KO mice (Postn−/−) (B6; 129-Postntm1Jmol/J) were purchased from the Jackson Laboratory (Bar Harbor, ME, USA). C57BL/6 J mice were obtained from Orient-Bio (Kyunggi-do, South Korea). The mice were housed in an animal facility and treated in accordance with the Guide for the Care and Use of Laboratory Animals of Seoul National University Hospital. All mice were housed under ambient conditions (standard humidity and temperature) with a 12 h light/dark cycle. The 7-week-old mice were used for experimentation after an adaptation period of 1 week. All mice were specific pathogen-free and were maintained under the same environmental conditions without differences in food intake.

### 2.2. Antibodies

The following primary antibodies were used in this study: periostin (ab14041; Abcam, Cambridge, MA, USA), alpha-smooth muscle actin (*α*-SMA: ab5694; Abcam, Cambridge, MA, USA), and collagen type 1 *α*2 (LS-C343921-100; LsBio, Seattle, WA, USA) for immunohistochemistry (IHC); connective tissue growth factor (CTGF: LS-B3284-50; LsBio, Seattle, WA, USA), transforming growth factor-beta (TGF-*β*: MAB240-100; R&D, Minneapolis, MN, USA), myeloperoxidase (MPO: AF3667; R&D, Minneapolis, MN, USA), vascular endothelial growth factor (VEGF: BS2431; Bioworld Technology, Louis Park, MN, USA), and *β*-actin (sc-47778; Santa Cruz Biotechnology, Inc., Dallas, Texas, USA) for western blotting. The following secondary antibodies were used: mouse anti-rabbit IgG-horseradish peroxidase (IgG-HRP: sc-2357; Santa Cruz Biotechnology, Inc., Dallas, Texas, USA), mouse anti-goat IgG-HRP (sc-2354; Santa Cruz Biotechnology, Inc., Dallas, Texas, USA), and rabbit anti-mouse IgG-HRP (ab6728; Abcam, Cambridge, MA, USA).

### 2.3. Implantation of the Silicone Implants

**Figure 1:**
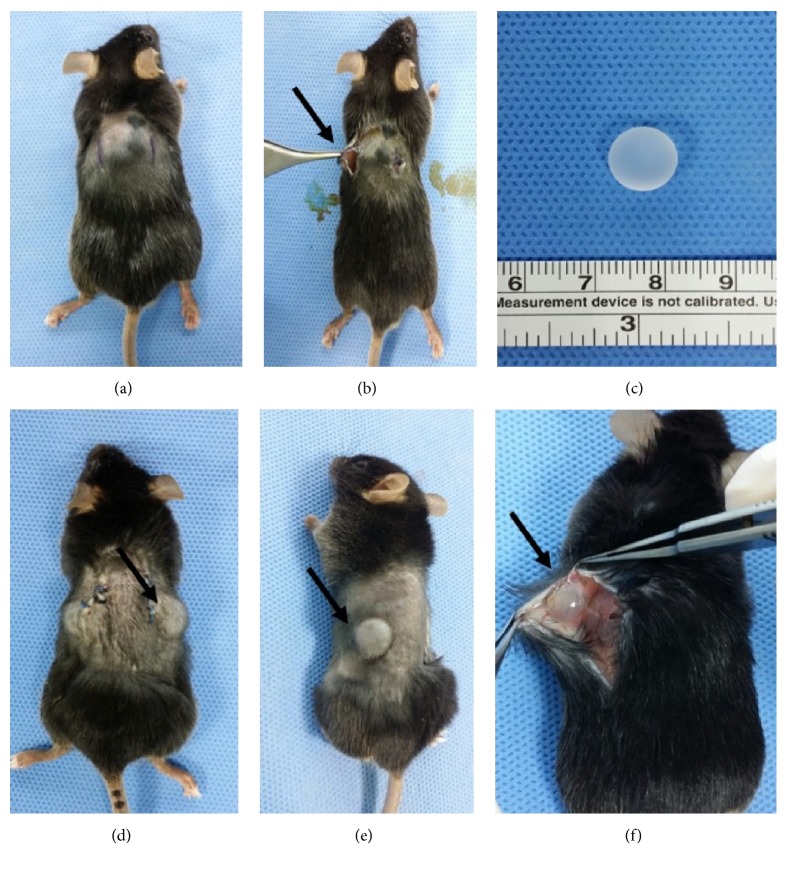
Implantation of the silicone implants (black arrows indicate the implantation site on the back). After two separate 2-cm vertical incision lines were made (a), subcutaneous pockets for implant insertion were formed on the back (b). Then, 0.8-cm-diameter hemispheric silicone implants (c) were inserted, and the surgical wounds were closed (d). After 8 weeks, the capsular tissue was harvested from the embedded silicone implants (e) and (f).

All surgical procedures were performed under aseptic conditions by a single surgeon (JUP). The C57BL/6 and PN-KO mice were each divided into two groups of six mice. Both groups received smooth silicone implants. The surgical field was prepared using 10% povidone-iodine, and a single dose of cefazolin (60 mg kg^−1^) was administered intramuscularly for prophylaxis of infection. The animals were anesthetized using an intraperitoneal injection of Zoletil (30 mg kg^−1^; JiWoo Pharm, Seoul, Republic of Korea) and Rumpun (5 mg kg^−1^; JiWoo Pharm, Seoul, Republic of Korea). Two subcutaneous pockets for implant insertion were made on the back of each mouse through two separate 2-cm vertical incisions, which were started at a lateral position 1.5 cm to the side of the midline and 1 cm below the shoulder bone (Figure 1). We used 0.8-cm diameter smooth-surfaced, solid hemisphere silicone implants, which were sterilized by autoclaving and exposure to ultraviolet light. The implants were inserted beneath the panniculus carnosus muscle. The surgical wounds were closed with successive layers of 4-0 Vicryl and 5-0 Ethilon (Ethicon, Inc., USA).

### 2.4. Harvest of Capsules from Embedded Silicone Implants

After 8 weeks, mice were sacrificed using CO_2_ asphyxiation in accordance with AVMA (American Veterinary Medical Association) Guidelines for the Euthanasia of Animals. The capsular tissue around the silicone implant was retrieved through the previously made incision (Figure 1). The harvested capsular tissues were fixed in 4% paraformaldehyde for at least 1 d and then embedded in paraffin. For immunohistochemistry, the extracted tissue was sectioned at a 4-*μ*m thickness. A portion of the harvested capsule was stored at −80°C for western blot analysis. The harvested capsule from the central portions of the upper and lower surfaces of the implant underwent gross examination.

### 2.5. Histological Analysis: Periostin, *α*-SMA, and Collagen Type I

The paraffin-embedded samples were mounted on coated slides, and after removing the paraffin, the slides were stained with Sirius red in saturated picric acid for 1 h at room temperature. After washing the slides with running tap water, hematoxylin was used to counterstain the nucleus for 1 min, followed by dehydration with 95% alcohol, clearing with dimethylbenzene, and gum mounting for H&E staining. Each stained slide was examined at ×100 magnification using a Leica DM2500 microscope (Leica Microsystems-Switzerland, Ltd, Switzerland), and images were captured from three microscopic fields: right, center, and left. Capsular thickness was measured at the maximal point using National Institutes of Health Image J 1.36b imaging software (National Institutes of Health, Bethesda, MD, USA). Thereafter, the cellularity was examined in each image. The number of cells per unit area was calculated automatically using the LAS Core Image Program (Leica Application Suite software, version 2.4.0; Leica Imaging Systems, Ltd, Cambridge, UK).

For IHC, tissue sections were blocked with phosphate-buffered saline (PBS) containing 0.15% Tween-20, 2% bovine serum albumin (BSA), and 5% normal donkey serum for 30 min at room temperature. Sections were then incubated with primary antibodies [rabbit polyclonal to periostin (1 : 500), rabbit polyclonal to *α*-SMA (1 : 400), and rabbit polyclonal collagen I alpha (COL1A1) (1 : 1,000)] in blocking solution overnight at 4°C. After washing three times in PBS, sections were incubated with species-specific HRP-conjugated secondary antibodies for 1.5 h at room temperature. Control sections were incubated with secondary antibody alone. Immunohistochemical staining was evaluated in three areas, as with H&E staining. *α*-SMA-positive cells that presented a brown color were manually counted in the unit area captured from three microscopic fields (right, center, and left), and the results are presented as the number of cells/mm^2^. The expression of collagen type I was measured as the total pixel intensity using Leica Q win image program V 3.2.0 (Leica Imaging Systems, Ltd), and the results are expressed as optical densities.

### 2.6. Western Blot Analysis: CTGF, TGF-*β*, MPO, and VEGF

Capsular tissues were solubilized by sonication in lysis buffer using PRO-PREP reagent (Intron Biotechnology, Daejeon, Republic of Korea), and the concentration of protein was measured using a BCA Protein Assay kit (Thermo-Fisher, Seoul, Republic of Korea). After being denatured by boiling, the protein sample (10 *μ*g for each lane) was separated by 12% sodium dodecyl sulfate-polyacrylamide gel electrophoresis and transferred to a polyvinylidene fluoride membrane (Millipore, Boston, MA). The blot was probed with a primary antibody [rabbit polyclonal to CTGF (C Terminus, IHC-plus™) (1 : 100)], mouse monoclonal to TGF-*β* (1 : 50), goat MPO antibody (1 : 1,000), rabbit polyclonal VEGF (1 : 1,000), or mouse monoclonal *β*-actin, (1 : 1,000)] in a blocking solution of 5% BSA in Tris-buffered saline containing Tween-20 (5% BSA-TBST) overnight at 4°C and then incubated with peroxidase-conjugated secondary antibodies (1 : 5,000) for 1 h at room temperature. The immunolabeled proteins were detected by chemiluminescence using a SuperSignal ECL kit (Pierce Chemical, Rockford, Ill) and ImageQuant LAS 4000 (GE Healthcare Life Science, Marlborough, MA, USA).

### 2.7. Statistical Analysis

All data are expressed as the mean ± standard error of the mean (SEM). Data analysis was performed using GraphPad Prism (version 7.0 for Windows; GraphPad Software, La Jolla, CA, USA). For all data, significant differences were determined using an unpaired *t*-test, assuming Gaussian distribution and that both populations had the same standard deviations. A *p* value of <0.05 was considered statistically significant, and the degree of difference is indicated in the figures as *∗∗* for *p* < 0.001 and *∗∗∗* for *p* < 0.0001.

## 3. Results

### 3.1. In Vivo Capsule Formation

**Figure 2:**
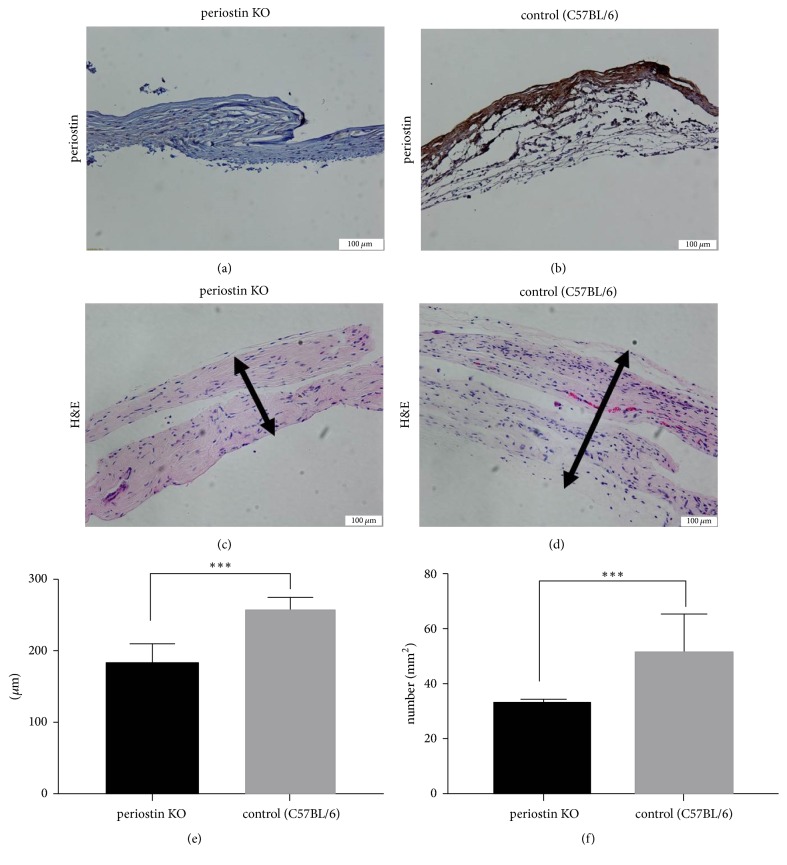
Expression of periostin (a) and (b) in silicone implant-induced capsular tissues by immunohistochemical analysis (original magnification: ×100). Hematoxylin-and-eosin-stained capsular tissue was observed under a light microscope (original magnification: ×200) (c) and (d). Capsular thickness (e) and the cellularity (f) were lower in the PN-KO group (*n* = 6) than in the control group (*n* = 6). Black arrows indicate capsular thickness. ^*∗∗∗*^*p* < 0.0001.

We designed an in vivo model as shown in Figure 1. We inserted silicone implants beneath the panniculus carnosus muscle on the back of mice so that we could observe capsule formation around the implants. After 8 weeks, tissues around the silicone implants were carefully collected in order to compare capsule formation. Immunohistochemical staining images of periostin revealed that periostin expression was considerably higher in the C57BL/6 control group than in the PN-KO group (Figures 2(a) and 2(b)). Initially, we compared the thickness of the capsules between the PN-KO and control C57BL/6 mice. Because periostin is known to accelerate fibrotic tissue formation, we speculated that it plays a role in silicone surface-induced capsule formation in vivo, which is considered to be a response to the presence of a foreign body. Histological estimation of the peri-implant capsular thickness showed significant differences between the PN-KO and C57BL/6 mice (Figures 2(c) and 2(d)). Capsular thickness was 193.6 ± 42.5 *μ*m in the PN-KO group and 258.5 ± 55.0 *μ*m in the control group (*p* < 0.0001, Figure 2(e)). We confirmed that the capsules in the experimental group were significantly thinner than those in the control group, indicating that capsule formation was significantly affected by periostin.

### 3.2. Cellularity

At 8 weeks after implantation, the PN-KO group (33.6 ± 12.7) showed a significantly lower cellularity per unit area than the normal control group (52.9 ± 25.8) (*p* < 0.001) (Figure 2(f)). The presence of increased number of inflammatory cells was indicative of the inflammatory phase, which represents the first step in capsule formation, whereby recruited inflammatory cells, such as neutrophils and macrophages, act as major mediators in inflammatory reactions by secreting various cytokines, recruiting fibroblasts and activating collagen synthesis.

### 3.3. Immunohistochemistry

**Figure 3:**
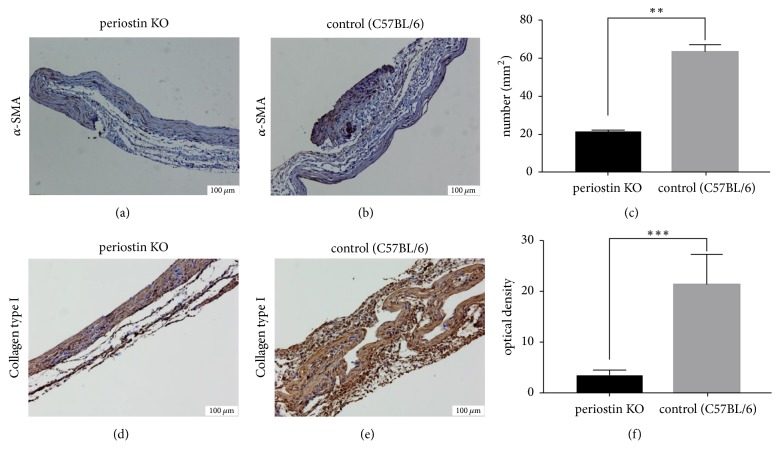
Immunohistochemical staining of *α*-SMA (a) and (b) and collagen type I (d) and (e) in silicone implant-induced capsular tissues by immunohistochemical analysis (original magnification: ×100). The number of *α*-SMA-stained cells was significantly lower in the PN-KO group (*n* = 6) than in the PN-KO group (*n* = 6) (c). The expression of collagen type I protein was significantly lower in the PN-KO group (*n* = 6) than in the control C57BL/6 group (*n* = 6) (f). ^*∗∗*^*p* < 0.001; ^*∗∗∗*^*p* < 0.0001.

IHC imaging for *α*-SMA and collagen type I was performed on sections of capsules formed around the silicone implants. We found that the *α*-SMA-expressing cell number was considerably higher in the C57BL/6 control group than in the PN-KO group (Figures 3(a), 3(b), and 3(c)). As shown in Figures 3(d), 3(e), and 3(f), there was also a significant reduction in collagen type I protein in PN-KO mice (*p* < 0.0001).

### 3.4. Western Blot Analysis

**Figure 4:**
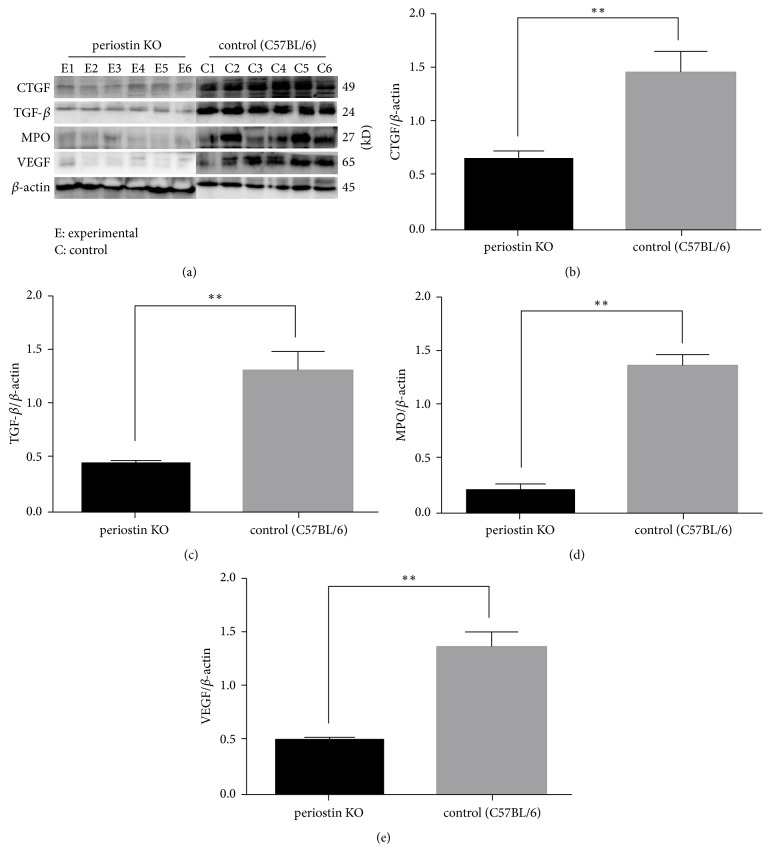
Levels of CTGF, TGF-*β*, MPO, and VEGF in silicone implant-induced capsular tissues determined by western blotting (a). A low signal was obtained for CTGF (b) and TGF-*β* (c) protein in PN-KO mice (*n* = 6), whereas a strong signal was detected in the C57BL/6 mice (*n* = 6). Compared with the control group (*n* = 6), the levels of MPO (d) and VEGF (e) protein were downregulated in the PN-KO group (*n* = 6). Relative expression levels normalized to the housekeeping gene *β*-actin. ^*∗∗*^*p* < 0.001.

To examine the function of periostin in the inflammatory reaction, collagen synthesis, and neoangiogenesis after silicone implant insertion, we monitored the expression of CTGF, TGF-*β*, MPO, and VEGF (Figure 4(a)). CTGF (Figure 4(b)) protein expression was lower in the PN-KO group compared with that in the C57BL/6 control group. TGF-*β* (Figure 4(c)) also showed a weak signal in the PN-KO group in contrast with the strong signal in samples from the C57BL/6 control group. These observations indicate that TGF-*β* is involved in capsule fibroblast differentiation as well as in aggravating the inflammatory phase and fibrosis, in accordance with the increase in CTGF expression. We also compared MPO levels (Figure 4(d)) in the capsular tissues between PN-KO and normal control mice using western blot analysis. As expected, tissue MPO levels were significantly reduced in the PN-KO group (*p* < 0.0001), indicating that blocking periostin may reduce the inflammatory signal during capsule formation, particularly that needed for inflammatory cell recruitment. In addition, we examined the levels of VEGF expression. As shown in Figure 4(e), VEGF expression (normalized to the housekeeping protein, *β*-actin) was a significantly downregulated in the experimental (PN-KO) group compared with that in the C57BL/6 control group. These observations indicate that blocking periostin inhibits capsular tissue activity such as the inflammatory reaction or neoangiogenesis.

## 4. Discussion

Although the cause of capsular contracture in breast implants is still controversial, there have been many attempts to explain the phenomenological mechanism [26]. Host reactions following the implantation of biomaterials such as breast silicone implants include five steps: blood/material interaction induced by vascularized connective tissue injury, early inflammation, late inflammation, granulation tissue development, and fibrosis/fibrous capsule development [27]. In the first step, a blood-based matrix layer forms around the biomaterial at the tissue/material interface. This step not only initiates inflammatory responses but also leads to thrombus formation involving the activation of platelets, which in turn releases TGF-*β* to attract inflammatory cells such as neutrophils (polymorphonuclear leukocytes: PMNs) [28]. Following this initial process, acute and chronic inflammation occur in a sequential cascade. PMNs characterize the early inflammatory response. The acute inflammatory response against biomaterials generally resolves quickly within less than 1 week. However, chronic inflammation persists due to the presence of mononuclear cells, that is, monocytes and lymphocytes, at the implant site for 3 weeks. In the fourth step, granulation tissue is formed with fibroblast proliferation and neovascularization. Granulation tissue is the precursor to fibrous capsule formation and is separated from the implant or biomaterial by the cellular components of the foreign body reaction: foreign body giant cells [29]. Fibrous capsules are formed in final step. Myofibroblasts are one of the predominant cell types involved in capsule formation [30–33]. According to Hwang et al. [34], the capsule consists of approximately 27% myofibroblasts, which increase the tensile strength according to the degree of contracture. Ashley et al. [35] previously showed that periostin expression was upregulated by the inflammatory response. Considering the relationship between periostin and myofibroblasts, previous studies have suggested that periostin promotes myofibroblast differentiation [23–25]. Treatment for reducing capsular contracture in breast implants remains speculative and multimodal: utilizing textured-surface breast implants [4], retromuscular implant placement [36], manual mobilization of the prosthesis (massage) [37], and local steroid injection [38]. However, there is no definite treatment with reliable results.

In recent years, studies on matricellular proteins related to capsular contracture have been conducted. Matricellular proteins are ECM proteins that modulate cell-matrix interactions as well as cellular functions. They are highly expressed in injured and remodeled tissues and have been implicated in the pathophysiology of various fibrotic conditions. Like other matricellular proteins, periostin is thought to play a fundamental role in tissue development and remodeling [39]. Using PN-KO mice, the importance of periostin in various fibrotic conditions has been unraveled. Yang et al. [25] used both PN-KO and WT mouse groups to elucidate the role of periostin in scleroderma by examining the downstream pathway involved in periostin signaling during the pathogenesis of scleroderma. The results indicated that periostin is involved in multiple steps of skin fibrosis (proliferation and recruitment of myofibroblasts and promotion of collagen assembly). In another study, Elliott et al. [40] assessed the contribution of periostin to dermal healing by comparing PN-KO and WT mice. They reported that full-thickness cutaneous wounds in PN-KO mice showed poor healing as a consequence of lower *α*-SMA expression in granulation tissue.

However, it has not been previously determined whether periostin is involved in the process of capsular contracture around biomaterials such as silicone breast implants. We hypothesized that regulation of the periostin associated with fibrosis is a key factor in mitigating capsular contracture. Based on our histological estimations, capsules around implants in the PN-KO group were significantly thinner than those around implants in the C57BL/6 group. Collagen type I expression was also significantly downregulated in the PN-KO group.

Capsules comprise a collagenous layer and noncollagenous layer. The external layer of the capsule, the collagenous layer, is composed of tissue rich in collagen, whereas the internal noncollagenous layer comprises synovial-like materials and loose conjunctive tissue [41]. Figure 2(e) shows that capsular thickness was decreased in the PN-KO group. In the collagenous layer, collagen synthesis occurs within a few hours of injury and progresses for months [42]. Periostin specifically mediates its effects on capsule formation by regulating collagen type I crosslinking [43]. In the noncollagenous layer, periostin may suppress the inflammatory cell response and thus reduce swelling and consequently decrease the thickness of this layer.

Our observations indicate that capsule formation is positively correlated with the degree of the inflammatory reactions. The PN-KO group clearly showed fewer inflammatory cells and inflammatory signals such as TGF-*β* and MPO at the 8-week time point than the control group. MPO has been proposed to mirror the degree of neutrophil activation [44]. Moreover, recent studies have suggested that periostin facilitates the infiltration of neutrophils, as the first cells recruited to the site of an allergic reaction in the airway [45]. Additionally, TGF-*β*, an important factor in the early inflammatory cascade, is a major cytokine secreted by several different cell types, such as platelets, giant cells, and fibroblasts. It eventually activates fibroblasts to promote collagen synthesis and stimulates the differentiation of fibroblasts into myofibroblasts [46]. Based on the results of the present study, we suggest that periostin promotes an increase in TGF-*β* levels after the implantation of silicone as a foreign body. In doing so, it can induce the activation of PMNs by controlling the process of the early inflammatory phase, via increasing the number of fibroblasts, which also increases the number of differentiated cells, that is, myofibroblasts in the 5th and final step of the host response.

We further analyzed CTGF, which is a cysteine-rich proadhesive matricellular protein that plays an essential role in the formation of connective tissue. CTGF is profibrotic, as it is overexpressed in fibrotic disease and synergizes with TGF-*β* to promote sustained fibrosis in vivo [47]. Mazaheri et al. [48] previously reported a positive relationship between increased CTGF levels and capsule formation. In the present study, CTGF density was significantly lower in the PN-KO group than in the control group. The activity of CTGF shows some similarities to that of TGF-*β*, in that it stimulates cell proliferation and ECM protein synthesis by fibroblasts.

One consequence of the protein cascade in capsule formation is neoangiogenesis, although there is still controversy as to whether neoangiogenesis aggravates capsular contracture [49]. VEGF is a potent angiogenic factor in vivo, and its activity represents an essential and rate-limiting step in physiologic angiogenesis [50]. In the present study, we found that the PN-KO group showed significantly lower VEGF expression than the C57BL/6 group. In the 4th step of the host response, periostin enhances the expression of VEGF associated with excessive formation of granulation tissue and accelerates new vessel formation.

It is widely accepted that *α*-SMA-expressing myofibroblasts, which are induced by fibrogenic cytokines, play key roles in collagen synthesis. Therefore, to determine whether periostin is required for myofibroblast differentiation in this model, we performed IHC analysis of *α*-SMA. We observed that the expression of *α*-SMA was significantly decreased in the PN-KO group. According to Yang et al. [25], periostin stimulation alone does not induce *α*-SMA expression in fibroblasts, but TGF-*β*-induced *α*-SMA expression could be enhanced by periostin. These results are consistent with our present findings. Taken together, periostin can indirectly control *α*-SMA expression through the activation of TGF-*β* in 5th and final step of the host response.

## 5. Conclusion

In the present study, we observed that PN-KO mice harboring silicone implants showed reduced in vivo peri-implant capsule formation. Periostin, an important protein in collagen synthesis and inflammatory processes, is considered to be essential for capsule formation. Accordingly, the inhibition of periostin could play an important role in suppressing capsule formation following the use of breast silicone implants in vivo.

## Abbreviations

WT: Wild-type
ECM: Extracellular matrix
*α*-SMA: Alpha-smooth muscle actin
CTGF: Connective tissue growth factor
TGF-*β*: Transforming growth factor-beta
IHC: Immunohistochemistry
MPO: Myeloperoxidase
VEGF: Vascular endothelial growth factor
HRP: Horseradish peroxidase
PN-KO: Periostin-knockout
PBS: Phosphate-buffered saline
BSA: Bovine serum albumin
TBST: Tris-buffered saline containing Tween-20
SEM: Standard error of the mean.
